# Movement and afferent representations in human motor areas: a simultaneous neuroimaging and transcranial magnetic/peripheral nerve-stimulation study

**DOI:** 10.3389/fnhum.2013.00554

**Published:** 2013-09-17

**Authors:** H. Shitara, T. Shinozaki, K. Takagishi, M. Honda, T. Hanakawa

**Affiliations:** ^1^Department of Functional Brain Research, National Center of Neurology and Psychiatry, National Institute of NeuroscienceKodaira, Japan; ^2^Department of Orthopedic Surgery, Gunma University Graduate School of MedicineMaebashi, Japan; ^3^Department of Orthopedic Surgery, Maki HospitalTakasaki, Japan; ^4^Department of Advanced Neuroimaging, Integrative Brain Imaging Center, National Center of Neurology and PsychiatryKodaira, Japan; ^5^PRESTO, Japan Science and Technology AgencyKawaguchi, Japan

**Keywords:** connectivity, motor cortex, multimodal neuroimaging, proprioceptive afferents

## Abstract

Neuroimaging combined with transcranial magnetic stimulation (TMS) to primary motor cortex (M1) is an emerging technique that can examine motor-system functionality through evoked activity. However, because sensory afferents from twitching muscles are widely represented in motor areas the amount of evoked activity directly resulting from TMS remains unclear. We delivered suprathreshold TMS to left M1 or gave electrical right median nerve stimulation (MNS) in 18 healthy volunteers while simultaneously conducting functional magnetic resonance imaging and monitoring with electromyography (EMG). We examined in detail the localization of TMS-, muscle afferent- and superficial afferent-induced activity in M1 subdivisions. Muscle afferent- and TMS-evoked activity occurred mainly in rostral M1, while superficial afferents generated a slightly different activation distribution. In 12 participants who yielded quantifiable EMG, differences in brain activity ascribed to differences in movement-size were adjusted using integrated information from the EMGs. Sensory components only explained 10–20% of the suprathreshold TMS-induced activity, indicating that locally and remotely evoked activity in motor areas mostly resulted from the recruitment of neural and synaptic activity. The present study appears to justify the use of fMRI combined with suprathreshold TMS to M1 for evoked motor network imaging.

## Introduction

For online control of movement, the status of muscles controlling the target effector needs to be properly monitored and integrated with a motor plan. Somatosensory afferents from the effector carry critical information for this somatosensory-motor integration. Such integration likely occurs at multiple levels in the central nervous system, from the spinal cord up through cortical motor areas. In primates, both superficial and proprioceptive sensations reach cortical motor areas including the primary motor cortex (M1) (Strick and Preston, 1978; Fetz et al., 1980; Lemon, 1981) presumably via the thalamus (Horne and Tracey, 1979) and primary somatosensory cortex (S1) (Pons and Kaas, 1986). We can therefore expect afferent-induced neural activity in motor areas during overt movement. Indeed, previous studies in humans have reported motor-area activity during peripheral nerve stimulation that elicits muscle-twitching (Korvenoja et al., 1999; Spiegel et al., 1999; Del Gratta et al., 2000; Shitara et al., 2011) and during vibrotactile stimulation to muscles (Naito et al., 2002; Gizewski et al., 2005; Bardouille and Ross, 2008). In contrast, few human neuroimaging studies have addressed the issue of motor-area activity ascribed to sensory afferents. This omission likely results from difficulty controlling the effects of sensory afferents during voluntary movement accompanying a complicated temporal pattern of muscle activity.

Neuroimaging combined with transcranial magnetic stimulation (TMS) to M1 is an emerging technique for non-invasively studying the organization of motor-control neural networks in humans. Suprathreshold TMS to M1 consistently induces increased brain metabolism or hemodynamic responses in the stimulated area as well as remote motor areas (Bestmann et al., 2003; Speer et al., 2003; Komssi et al., 2004; Fox et al., 2006; Hanakawa et al., 2009; Shitara et al., 2011). However, because suprathreshold TMS induces motor evoked potentials (MEPs) visible on surface electromyography (EMG), we must consider the effects of proprioceptive afferents from the twitching muscles when interpreting such motor-area activity.

Technical advances now allow us to measure MEPs with surface EMG in a simultaneous TMS-fMRI (functional magnetic resonance imaging) environment (Bestmann et al., 2004; Hanakawa et al., 2009; Shitara et al., 2011). An MEP is composed of a brief oligophasic potential similar in shape to a compound muscle action potential (CMAP) evoked by peripheral nerve stimulation. Motor-area activity during peripheral nerve stimulation, if any, should reflect processing of sensory afferents without preceding motor commands. Given the similarity of MEPs and CMAPs, using peripheral nerve stimulation as a control condition for muscle afferents during suprathreshold M1 stimulation seems reasonable. However, MEPs are always smaller than CMAPs because of phase cancellation of action potentials at the corticospinal and spinal neuron levels (Magistris et al., 1998). Still, CMAPs and MEPs should provide rough yet comparable estimates of proprioceptive afferent size because the phase cancellation occurs at the motor efferent level, not at the muscular or afferent level. Additionally, peripheral nerve stimulation between the motor and sensory thresholds can provide a condition akin to superficial sensory stimulation.

Based on these assumptions, here we conducted a multimodal imaging study to differentiate neural activity in the motor network that generates movement from those used for afferent information processing resulting from the movements. The main goal was to compare local and remote brain activity resulting from suprathreshold single-pulse TMS to M1 with those resulting from electrical median nerve stimulation (MNS) above the motor threshold. In particular, we were interested in assessing TMS-evoked and sensory-evoked activity in M1 subdivisions (M1a and M1p) that are reported to have a differential distribution of proprioceptive and superficial afferent information (Strick and Preston, 1982; Geyer et al., 1996).

## Materials and methods

### Subjects

We analyzed data from 18 healthy adults (female, 15; male, 3; mean age, 29.3 years; range, 20-46 years) in our previous study (Shitara et al., 2011). From this previous study, we selected participants who underwent both the TMS and MNS conditions in the same experimental session. All participants were right handed. None reported any history of neuropsychiatric disorders, including epilepsy. The Institutional Review Board of the National Center of Neurology and Psychiatry approved the study protocol. The participants were fully informed about the experimental procedure, and all gave written informed consent prior to the participation.

### Stimulation and electromyography monitoring

We used a 3-Tesla whole-body MRI scanner equipped with a circular polarization head coil (Siemens Magnetom Trio; Erlangen, Germany). The “motor hot spot” at which TMS evoked a maximal motor response in the right abductor pollicis brevis (APB) muscle was identified for each participant while lying supine on the scanner bed. The APB was the primary muscle of interest in this experiment, as with our previous TMS-fMRI experiments (Hanakawa et al., 2009; Shitara et al., 2011). An MRI-compatible figure-eight TMS coil with an outer-wing diameter of 70 mm (MR coil, Magstim, Witland, Wales, UK) was positioned tangentially to the scalp at the “motor hot-spot.” TMS-coil orientation was ~45° from the medial-lateral axis. The TMS coil was connected to a stimulator (SuperRapid, Magstim, Witland, Wales, UK) via a 7-m cable running through a wave-guide tube appropriate for radiofrequency wave filtering. The TMS stimulator produced a biphasic electrical lasting ~250 μs with a rise time of 50 μs.

For the MNS-fMRI experiment, electrical stimulation was delivered through a pair of MRI-compatible silver/silver chloride electrodes (Nihon Kohden, Tokyo, Japan). The electrodes were connected to an electric current stimulator (Nihon Kohden, Tokyo, Japan) placed outside the scanner room. The maximum stimulator output was 50 mV. Constant-voltage square waves with pulse duration of 0.3 ms were applied to the right median nerve at the wrist. The sensory threshold was determined by each participant's verbal report of sensation in the first three fingers without muscle twitching. A motor threshold for eliciting APB activity was defined according to the recommendations of the IFCN Committee (Rossini et al., 1994a) as the minimum stimulus intensity that produced a liminal EMG response (more than 50 mV in 50% of trials). No participants reported a sensation of pain. The stimulation procedure did not cause any artifacts in the functional MR images.

MEPs were recorded from the APB and abductor digiti minimi (ADM) muscles bilaterally, using BrainAmp ExG MR (Brain Products, Gilching, Germany). The amplifier and MRI scanner were synchronized using SyncBox (Brain Products, Gilching, Germany). Surface electrodes were placed over the bilateral APB and ADM muscles with an inter-electrode distance of ~2 cm. EMG signals were fed into a battery-driven amplifier placed on the scanner bed. EMG data were sampled at a digitization rate of 5 kHz with an amplitude resolution of 0.5 μV/bit and a dynamic range of 16 mV.

To minimize joint movements and reflexive antagonistic muscle contraction, both upper limbs were tightly fixed onto custom-made, non-magnetic splints (covering the hand, wrist, and elbow joints) with elastic bandages. Hence, both TMS- and MNS-evoked movements were primarily isometric contractions. Fixing the hand position also helped minimize EMG changes and imaging artifacts throughout the experiment. The position of the TMS coil was adjusted while stimulation was delivered every 5 s until MEPs were consistently recorded from the right APB muscle. The TMS coil was then fixed immobile to the scanner bed with a custom-made holder made from polyetheretherketone plastic. Foam pads and vacuum cushions were used to minimize head motion during scanning. After participants' heads were fixed, the resting motor threshold (RMT) was defined individually as the percentage of stimulator output that elicited MEPs of greater than 50 μ V peak-to-peak amplitude in the APB at rest in more than 5 of 10 successive trials (Rossini et al., 1994a). The active motor threshold (AMT) was similarly determined during weak isometric contraction of the APB muscle at ~20% of the maximum contraction under EMG monitoring.

### Experimental tasks

During the fMRI experiments, participants were instructed to relax and remain awake. Vision was not constrained. In each fMRI run, 42 single-pulse TMS or MNS were given during 110-ms inter-volume acquisition delay periods. Stimulus onset asynchrony (SOA) was semi-randomized between 7.98 and 13.97 s (stimulus frequency, 0.072-0.125 Hz). All participants underwent the TMS-fMRI and MNS experiment. The experimental conditions were composed of suprathreshold TMS at an intensity of 120% of the RMT (supra-TMS), MNS above the motor threshold (motor-MNS), and MNS between the sensory and the motor thresholds (sensory-MNS). The sensory-MNS condition was included to account for the sensory-component stimulation of the MNS condition and to map representations of non-muscular afferents to motor areas. Stimulus intensity during motor-MNS varied semi-randomly between the motor threshold and 120% of the motor threshold to yield variability similar in evoked movement size as in the supra-TMS condition. Intensity of sensory-MNS was kept between the motor and the sensory thresholds for all participants and was not varied.

Stimulation timing was controlled by Presentation software (Neurobehavioral systems, Albany, CA, USA) on a personal computer synchronized with the MRI scanner via transistor-transistor logic pulses converted from the default optic signals of the scanner. To avoid image degradation, TMS pulses were delivered during the inter-volume delay periods. The same stimulation timing was used for all conditions.

### Image acquisition

We measured hemodynamic signals with echo planar imaging (EPI) as follows: repetition time (*TR*) = 998 ms, inter-volume acquisition delay = 110 ms, echo time (*TE*) = 25 ms, flip angle (*FA*) = 60°, 64 × 64 matrix, 12 slices, 500 volumes, field of view (*FOV*) = 192 mm, and 3 × 3 × 4-mm voxel size. Bilateral cortical motor and somatosensory areas, basal ganglia, and thalami were covered with approximately coronal acquisition. Most of the cerebellum was outside the search volume. A short TR was employed originally to investigate the signal time-course of TMS-evoked activity with fine temporal resolution (Shitara et al., 2011). The timing of gradient pulses was carefully designed and the MRI scanner and amplifier were synchronized so that EMG signals could be constantly sampled at 1 kHz without being disturbed by gradient pulses (Anami et al., 2003). The inter-volume acquisition delay allowed us to acquire EPI without interference from induced electromagnetic fields or vibration of the TMS coil.

For anatomic registration, T1-weighted three-dimensional structural images were also acquired with a magnetization-prepared, rapid-gradient echo sequence (*TR* = 2000 ms, *TE* = 4.38 ms, *FA* = 8°, *FOV* = 192 mm, matrix = 176 × 192, voxel size = 1 × 1 × 1 mm). For correcting distortion of functional images, field-map imagers were obtained in the same space as the functional image (*TR* = 511 ms, *TE* = 5.19 and 7.65 ms, *FA* = 60°, *FOV* = 192 mm, matrix = 64 × 64, voxel size = 3 × 3 × 4 mm).

### EMG and image data analysis

To remove gradient artifacts, we pre-processed all EMG data using an averaged subtraction method implemented in Analyzer 2 (Brain Products, Gilching, Germany). A band-pass filter of 20-200 Hz was applied. We visually checked the quality of artifact-removed EMG data from the target (right APB) for quantitative assessment of right APB activity and those of non-target (left APB and bilateral ADM) muscles to confirm the absence of EMG. The artifact-free EMG data from the right APB were rectified and integrated to produce an integral EMG value (iEMG) as a parameter of movement size. iEMG is adequate for quantifying total muscle activity with surface EMG (Hermens et al., 1991; Shitara et al., 2011) especially because we aimed to estimate the amount of muscle afferents in two types of evoked movements (MEP and CMAP) with slightly different temporal profiles. These different temporal profiles result from larger temporal jittering in TMS-evoked descending volleys than MNS-induced peripheral nerve excitation. Thus, it would be difficult to estimate the size of efferent signals by comparing iEMG between different modalities. However, iEMG can still serve as a surrogate marker for the quantity of afferent signals from the twitched APB. For computing the iEMG, time windows for the integration were modified to extract EMG signals specific to experimental conditions as purely as possible. Time for integration was between 20-40 ms for TMS and between 5–25 ms for the MNS (Shitara et al., 2011).

Two parameters were computed from the iEMG. First, mean iEMG was defined as the average of raw iEMG values divided by the EMG-evoking events in an fMRI run for the supra-TMS and the motor-MNS conditions. Mean iEMG data were compared across conditions with a paired *t*-test. Second, the minimum and maximum iEMG values across all EMG-evoking events were determined for each individual. Then, normalized iEMG was computed individually for each EMG-evoking event as follows: (iEMG - minimum iEMG)/(maximum iEMG - minimum iEMG). Normalized iEMG represented the variation of iEMG values across all the EMG-evoking events and served a parametric modulator in the first-level general linear model (GLM) analysis.

Imaging data were pre-processed with SPM5 (Wellcome Department of Imaging Neuroscience, UCL, London, UK) using Matlab (MathWorks, Inc., Natick, MA, USA) and FSL (FMRIB, Oxford University, Oxford, UK) using VMware player (VMware, Palo Alto, CA, USA). The first 10 volumes in each experimental run were discarded to allow for T1 equilibrium effects. The remaining functional images were corrected for differences in slice acquisition timing. The non-linear distortion of EPI data resulting from inhomogeneity of the magnetic field was corrected using FUGUE (FSL) by referencing to the field map image acquired for each participant before the fMRI experiment. The functional images were motion-corrected, and residual noise was detected with an independent component-analysis filter (MELODIC, FSL). We removed ICA components with time-course that showed abrupt spikes and those with maps showing alternating patterns of signal variation for every other slice (corresponding to motion during interleaved MRI data acquisition) (Tohka et al., 2008). The motion-corrected and artifact-removed images were then spatially normalized to fit to the Montreal Neurological Institute (MNI) template based on the standard stereotaxic coordinate system. Subsequently, all images were smoothed with an isotropic Gaussian kernel of 8-mm full-width at half-maximum.

Statistical analysis was performed using SPM5. A train of delta functions representing stimulus onsets was convolved with the canonical hemodynamic response function (HRF) and its temporal derivative, and these served as regressors for each condition (supra-TMS, motor-MNS, and sensory-MNS) in the first-level GLM analysis. Although neural events associated with these conditions may differ in the range of several tens of milliseconds, our previous study investigating fine temporal time-course of fMRI responses has clearly showed that we can estimate size of brain activity using the same HRF function for the three conditions (Shitara et al., 2011). We built two types of first-level design matrix: one with the normalized iEMG regressor as a parametric modulator of the EMG-evoking events and the other without. The latter design was used for analysis that included the sensory-MNS condition, which did not evoke muscle activity. Six parameters representing the head motion were included in the design matrix as covariates. Global signal normalization was performed only between runs. Low frequency noise was removed with a 128-s high-pass filter, and serial correlations were adjusted using an auto-regression model. We computed summary images reflecting the effects of interest on fMRI signals by applying linear contrasts to the parameter estimates. These summary images were fed into the subsequent second-level random-effect model analysis.

First, group-level statistical parametric maps (SPMs) were generated by performing one-sample *t*-tests on the summary images representing the effects of each experimental condition relative to the implicit baseline. Second, we defined proprioceptive and superficial sensory-evoked brain activity using the motor-MNS and sensory-MNS conditions. Muscle proprioception-evoked activity was defined by the difference in activation between the motor-MNS and sensory-MNS conditions (motor-MNS minus sensory-MNS). To define areas related to superficial afferent processing, a conjunction analysis was performed between the motor-MNS and sensory-MNS conditions since both types of stimulation should involve superficial afferents. Furthermore, we explored variation of brain activity explained by the size variation of the normalized iEMG regressor.

In the group-level SPM, the threshold was initially set at a voxel-wise height-level of *P* < 0.05 corrected for multiple comparisons (family-wise error; FWE). Clusters exceeding a height threshold of uncorrected *P* < 0.005 were reported as a trend. The cytoarchitectonic nomenclature of significant brain activity was identified according to the SPM5 anatomy toolbox when applicable.

### Distribution of TMS- and afferent-induced brain activity in M1 subdivisions

Previous animal and human studies have indicated differences in proprioceptive and superficial afferent information coded in M1 (Strick and Preston, 1982; Geyer et al., 1996). We therefore assessed the difference in spatial distribution between TMS- and two afferent-induced activities in M1 subdivisions (M1a and M1p). We created M1a and M1p mask-images (SPM5 anatomy toolbox) according to the MNI coordinates. The transformed M1a and M1p images were thresholded at 40% of their probabilistic distributions, and were then transformed into binary mask images (M1a and M1p masks). Brain activity obtained from the conjunction analysis of motor-MNS and sensory-MNS conditions was defined as the activity coding superficial afferents. Muscle afferent-related activity was defined as the contrast motor-MNS minus sensory-MNS (as described above). The “pure” TMS-induced activity was defined as the contrast supra-TMS minus muscle afferent-related activity to exclude the contribution of sensory components. In this particular analysis, we will report significant activities thresholded at *P* < 0.05 (FWE-corrected) after small volume correction according to our previous reports (Hanakawa et al., 2009; Shitara et al., 2011).

We also used small-volume correction (svc) analysis with spherical volumes of interest (VOIs). We applied a 5-mm radius to left M1 (*x, y, z* = −36, −24, 52), right M1 (*x, y, z* = 34, −30, 64), left dorsal premotor cortex (*x, y, z* = −32, −10, 60), left S1 (*x, y, z* = −32, −32, 68), left supplementary motor area (SMA) (*x, y, z* = −6, −12, 48), left putamen (*x, y, z* = −30, −10, 0), and left thalamus (*x, y, z* = −14, −22, 4), and a 3-mm radius to the left subthalamic nucleus (*x, y, z* = −12, −18, −4) (Hanakawa et al., 2009; Shitara et al., 2011). We also report trends toward activation thresholded at uncorrected *P* < 0.005.

### Distribution of TMS-induced and afferent-induced activity in M1subdivisions

We evaluated the spatial distribution of afferent-related activity and “purely” TMS-evoked activity represented in M1 subdivisions. We measured the total number of suprathreshold voxels at uncorrected *P* < 0.005 for “purely” TMS-evoked activity, muscle afferent-related activity, and superficial afferent-related activity within the M1a and M1p masks. To semi-quantify the differences in muscle- and superficial-afferent representations within the M1 subdivisions, an M1 divisional index (M1-DI) was calculated as follows: *DI* = (*V*_M1a_ − *V*_M1p_)/(*V*_M1a_ + *V*_M1p_), where V_M1a_ and V_M1p_ refer to the number of activated voxels within the M1a and M1p masks, respectively. DI ranged from −1.0 to 1.0. A positive DI value indicates that the activity is distributed disproportionately in M1a and a negative DI means that the activity is distributed disproportionately in M1p. Additionally, we computed DI for supra-TMS induced activity to test its distribution in the M1 subdivisions. A non-parametric statistic was applied to assess differences in DI for muscle afferents and superficial afferents. To gain knowledge about spatial distribution of TMS-induced activity within M1 subdivisions, we also computed DI for voxels related to the “pure” TMS.

### Removal of muscle afferent-induced activity corrected for movement size from TMS-induced activity in motor areas

By controlling differences in muscle activity across conditions, we were able to explore supra-TMS-evoked activity that could not be explained by sensory afferent responses to twitching movements. Supra-TMS and motor-MNS induced activities were corrected using iEMG size as a parametric modulator. The corrected activities are referred to as supra-TMS_iEMG_corr_. and motor-MNS_iEMG_corr_., respectively. Data from 12 participants were available for this comparison of motor MNS- and TMS-evoked brain activities. fMRI signals (beta values) were extracted from the motor area VOIs (as described above; left M1a mask, left M1p mask, left premotor cortex, and left SMA) using MarsBaR (Brett et al., 2002) for the contrast that compared motor MNS- and TMS- induced brain activity with and without iEMG correction. We then calculated how much of the activity during the supra-TMS condition could be ascribed to responses induced by the muscle afferents and how much could be explained purely by TMS with and without correction for iEMG differences. For this purpose, muscle afferent-induced activity was defined as motor-MNS minus sensory-MNS as above. “Pure” TMS-induced activity was defined as supra-TMS activity minus muscle afferent-induced activity. We computed these values with and without correction for iEMG size during the supra-TMS and motor-MNS conditions.

## Results

### TMS intensity and mean iEMG values

The mean RMT was 80.6% (*SD* = 8.7) of the machine output within the MRI scanner. Accordingly, the mean stimulus-intensity for the supra-TMS condition was 96.7% (10.4). For participants who required stimulation above 100% of the machine output to reach the 120% RMT stimulation, the maximum machine output of the TMS stimulator was reset to 110% of the default machine output (enhanced mode), which was within the safety guidelines of the manufacturer and its distributor in Japan (Magstim and Miyuki Giken). All participants completed the planned experimental conditions, and none experienced significant adverse events.

We visually checked artifact-removed EMG data to confirm sufficient data quality for quantitative analysis. As reported previously (Shitara et al., 2011), muscle activity was only observed in the right APB muscle in both supra-TMS and motor-MNS conditions. We did not find any muscle activity in the bilateral ADM or the left APB muscle(s). This finding indicated that TMS-evoked muscle activity did not spread to neighboring muscles. However, EMG data from six subjects were excluded from the iEMG analysis in the motor-MNS condition because artifacts substantially overlapped with CMAP. Hence, only data from 12 participants were available for the comparison between the motor MNS- and TMS-evoked brain activities that used iEMG as a parametric modulator. The mean iEMG expressed in mV × ms was 5.0 (*SEM* = 1.2) in the supra-TMS condition, and 34.7 (6.8) in the motor-MNS condition. A paired *t*-test showed a significant difference in iEMG across conditions (*P* < 0.0001).

### Muscle afferent-related activity: comparison between motor-MNS and sensory-MNS

**Figure 1:**
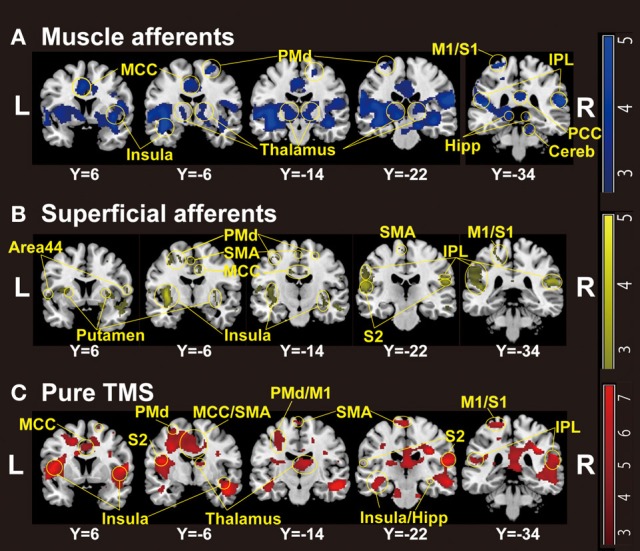
**Group-level statistical parametric maps (*n* = 18) showing the categorical comparison of conditions (thresholded at *P* < 0.005 uncorrected for display purpose). (A)** Muscle afferent-related activity (contrast, motor-MNS minus sensory-MNS). Brain activation was observed in bilateral insula, middle and posterior cingulate cortices, bilateral thalami, bilateral dorsal premotor cortices, bilateral hippocampi, bilateral inferior parietal cortices, right cerebellum, left M1, and left S1. **(B)** Superficial afferent-related activity (conjunction, motor-MNS and sensory MNS). Brain activation was observed in bilateral Area 44, bilateral putamen, bilateral insula, middle cingulate cortex, bilateral dorsal premotor cortices, SMA, bilateral S2, bilateral inferior parietal cortices, left M1, and left S1. **(C)** Supra-TMS-induced activity after removing the effects of muscle afferent-induced activity [contrast, (supra-TMS minus implicit baseline) minus (motor-MNS minus sensory-MNS)]. Brain activation was observed in bilateral insula, middle cingulate cortex, SMA, bilateral thalami, bilateral hippocampi, bilateral S2, bilateral inferior parietal cortices, left dorsal premotor cortex, left M1, and left S1. *MCC*, middle cingulate cortex; *PCC*, posterior cingulate cortex; *SMA*, supplementary motor cortex; *PMd*, dorsal premotor cortex; *M1*, primary motor cortex; *S1*, primary somatosensory cortex; *S2*, secondary somatosensory cortex; *IPL*, inferior parietal lobule; *Hipp*, hippocampus; *Cereb*, cerebellum.

Whole-brain analysis revealed significant activation related to muscle afferents in bilateral superior temporal gyri, bilateral insula, left middle cingulate cortex, left postcentral gyrus, and right supramarginal gyrus at the formal threshold (*P* < 0.05, FWE-corrected). After small volume correction, significant activation was observed in left precentral gyrus, left SMA, left putamen, left thalamus, and the subthamamic nucleus at a threshold of *P* < 0.05, FWE-corrected. Trends were found in the right inferior frontal gyrus, right inferior temporal gyrus, and left inferior parietal lobule (*P* < 0.005 uncorrected) (Figure 1A; Table 1A).

**Table 1:** **Results of group-level statistical parametric mapping analysis**.

*****N*** = **18****	**Coordinates (mm)**			***Z*-values**
**Activity clusters (functional anatomy)**	***x***	***y***	***z***	
**(A) MOTOR-MNS MINUS SENSORY-MNS (UNCORRECTED ***P*** < **0.005**)**				
Left superior temporal gyrus (Insula)	−40	−6	−12	5.06^*^
Right superior temporal gyrus (IPL)	58	−34	20	4.71^*^
Right supramarginal gyrus (OP1, IPL)	62	−22	22	4.55^*^
Left middle cingulate cortex	−6	10	34	3.84^*^
Left postcentral gyrus (S1)	−30	−34	72	3.81^*^
Right superior frontal gyrus (SMA)	20	−10	60	3.82
Left subthalamic nucleus	−12	−20	−2	3.63^#^
Left thalamus	−12	−22	0	3.59^#^
Left SMA	−4	−10	44	3.51^#^
Left putamen	−34	−12	−2	3.33^#^
Left precentral gyrus (M1)	−32	−32	68	3.29^#^
Right inferior frontal gyrus (Area 44)	40	10	30	2.89
Left precentral gyrus (M1)	−22	−28	54	2.87
Left precentral gyrus (S1, M1)	−24	−36	50	2.77
Right inferior temporal gyrus	44	−12	−28	2.67
Left inferior parietal lobule (S1)	−50	−38	56	2.62
**(B) CONJUNCTION OF MOTOR-MNS AND SENSORY-MNS (UNCORRECTED ***P*** < **0.005**)**				
Left insula (OP3)	−38	−8	2	5.71^*^
Left superior temporal gyrus (OP1)	−64	−28	16	5.14^*^
Right superior temporal gyrus	52	−36	22	5.03^*^
Left putamen	−34	−8	−2	5.03^#^
Right rolandic operculum (OP1)	56	−22	22	4.39^*^
Right temporal pole-Insula	46	2	−18	4.27^*^
Right inferior frontal gyrus (Area 44)	50	14	14	4.11^*^
Right temporal pole	50	10	−22	3.90^*^
Left postcentral gyrus (M1/S1)	−28	−36	64	3.58
Left postcentral gyrus (S1)	−16	−34	50	2.96
Left middle cingulate cortex	−12	−14	34	3.48
Right middle cingulate cortex	10	−6	38	3.27
Left precentral gyrus (PMd)	−30	−8	54	3.25
Left precentral gyrus (PMd/M1)	−40	−14	52	2.84
Left SMA	−2	−6	52	3.08
Left paracentral lobule (SMA)	−6	−22	66	2.97
Right precentral gyrus (PMd/Area 44)	52	10	40	3.09
Right PMd	20	−12	56	3.00
Right middle frontal gyrus	28	10	50	3.00
Left SMA	−4	−8	50	3.00^#^
Left SPL	−20	−40	40	2.70
Right thalamus	4	−20	−6	2.59
Left precentral gyrus (M1)	−38	−20	54	2.47^#^
**(C) (SUPRA-TMS GREATER THAN MOTOR-MNS) MINUS SENSORY-MNS (UNCORRECTED ***P*** < **0.005**)**				
Right middle temporal gyrus	52	−14	−16	5.27^*^
Right insula	46	−4	−22	4.88^*^
Right inferior frontal gyrus (Area 44)	48	14	4	4.87^*^
Right SPL	34	−32	34	4.89^*^
Left insula	−46	4	6	4.85^*^
Right PMd	34	−24	32	4.66^*^
Right cerebellum	18	−24	−30	3.86
Left paracentral lobule (M1)	−16	−32	72	3.83
Left paracentral lobule (SMA/M1)	−4	−22	72	3.50
Left PMd	−30	−10	58	3.16
Left M1	−18	−30	52	3.14
Left Hippocampus	−42	−24	−14	3.77
Left middle temporal gyrus	−46	−8	−22	3.39
Left S1	−28	−32	70	3.33^#^
Left superior temporal gyrus (OP1)	−60	−30	14	3.70
Left IPL	−40	−38	22	3.18
Left M1	−32	−32	68	3.03^#^
Right middle frontal gyrus	38	4	58	2.76
Left SMA	−4	−8	46	2.73^#^
Left putamen	−34	−8	2	2.41^#^
Left M1	−34	−20	50	2.35^#^
Right parahippocampal gyrus (Hippocampus)	22	−38	−10	2.65
Right superior frontal gyrus (SMA)	18	6	66	2.64
Right hippocampus	32	−20	−10	2.61

* FWE P < 0.05.

# P < 0.05 (FWE-corrected) after small volume correction.

M1, primary motor cortex; S1, primary somatosensory cortex; SPL, superior parietal lobe; hIP, intraparietal sulcus; OP, parietal operculum/secondary somatosensory cortex; IPL, inferior parietal lobule; TE, auditory cortex; Area 44, 45, Broca's areas.

### Brain areas related to superficial afferents

Significant activation related to superficial afferents was found in left insula, bilateral superior temporal gyri, right Rolandic operculum, right temporal pole, and inferior frontal gyrus at the formal threshold (*P* < 0.05, FWE-corrected). After small volume correction, significant activation was observed in left precentral gyrus, left SMA, and left putamen at the threshold of *P* < 0.05, FWE-corrected. Trends toward activation (*P* < 0.005 uncorrected) were found in the left postcentral gyrus, right precentral gyrus, left paracentral lobule, bilateral middle cingulate cortices, left supraparietal lobule, right middle frontal gyrus, and right thalamus (Figure 1B; Table 1B).

### Comparison between supra-TMS and muscle afferent-induced activity without correction for iEMG size

Because iEMG during motor-MNS was significantly larger than that during supra-TMS, a simple subtraction of muscle afferent-related activity from supra-TMS-induced activity would overcorrect the muscle afferent-related components in the supra-TMS-induced activity. Hence, although the components evoked by supra-TMS should be underestimated, the contrast supra-TMS > motor-MNS minus sensory-MNS revealed significant activation in the bilateral insula, right inferior frontal gyrus, right supraparietal lobule, and right middle temporal gyrus (*P* < 0.05 FWE-corrected). After small volume correction, significant activation was observed in the left pre/post central gyrus, left SMA, and left putamen at the threshold of *P* < 0.05 FWE-corrected. Trends toward activation were found in the left paracentral lobule (M1 and SMA), left middle-superior temporal gyrus, right middle-superior frontal gyrus, bilateral hippocampi, and right cerebellum (Figure 1C; Table 1C).

### Comparison between supra-TMS and muscle afferent-induced activity with correction for iEMG

Because motor-MNS produced larger muscle activity than supra-TMS, simple subtraction of muscle afferent-induced activity from supra-TMS-induced activity should overcorrect the effects of muscle afferents included in the supra-TMS-induced activity. We therefore reanalyzed data from the twelve participants for whom iEMG values were available for both supra-TMS and motor-MNS conditions. Specifically, to correct for differences in iEMG sizes, we used iEMG values as a parametric modulator of stimulation events for supra-TMS and motor-MNS. The contrast supra-TMS minus motor-MNS-corrected-for-iEMG revealed significant activity in left M1, left SMA, and left PMd (*P* < 0.05 FWE-corrected for small volume within VOIs) according to our previous reports (Hanakawa et al., 2009; Shitara et al., 2011) (Table 2). Trends toward activation were found in left postcentral gyrus (S1), middle frontal gyrus, bilateral precentral gyri (PMd), bilateral Rolandic operculum, bilateral insula, bilateral inferior temporal gyri, right thalamus, right SMA, left paracentral lobule, bilateral superior temporal gyri, left middle temporal gyrus, left inferior parietal lobule, and right MCC. Less prominent activity compared to a similar analysis without iEMG correction (described above) resulted from a lower degree of freedom. In fact, when the data from the same twelve participants were reanalyzed without using the iEMG parameter (Table 2), the iEMG-corrected analysis tended to yield greater *Z* values than the non-iEMG-corrected version. This finding supports the notion that a simple comparison between supra-TMS and motor-MNS would have underestimated “pure” TMS-induced activity. Therefore, the non-iEMG corrected analysis above is conservative in claiming that muscle afferents alone cannot explain motor-area activity during supra-TMS delivered to M1 (Figure 2; Table 2).

**Table 2:** **Supra-TMS compared with motor-MNS (Uncorrected *P* < 0.005)**.

*****N*** = **12****	**Functional anatomy**	**Coordinates (mm)**			**iEMG correction**	
**Activity clusters**		***x***	***y***	***z***	**(+)**	**(−)**
					***Z*-values**	***Z*-values**
Left middle frontal gyrus	PMd	−24	−6	48	3.75	NA
Left precentral gyrus	PMd	−32	−8	58	3.43^#^	NA
Left rolandic operculum		−44	−2	16	3.44	NA
Left insular lobe	Insula	−34	2	14	3.11	NA
Right inferior temporal gyrus		52	−4	−34	3.38	3.19
Left inferior temporal gyrus		−44	0	−32	3.23	NA
Right thalamus		24	−28	12	3.15	NA
Right SMA		10	8	52	3.05	NA
Left postcentral gyrus	S1	−18	−32	74	3.05	3.00
Left paracentral lobule	M1a, S1, SMA	−10	−32	72	2.82	2.80
Right superior temporal gyrus	IPL, OP1	60	−34	10	2.91	NA
Left middle temporal gyrus		−56	4	−18	2.83	NA
Right precentral gyrus	PMd	42	−2	38	2.82	NA
Left paracentral lobule	M1a, SMA	−8	−28	74	2.79	2.66
Left inferior parietal lobule	SPL	−36	−34	38	2.76	NA
Right insula lobe	Insula	36	−20	6	2.68	NA
Left superior temporal gyrus	TE1	−50	−24	10	2.62	NA
Right rolandic operculum	Area44	54	10	0	2.60	2.64
Right middle cingulate cortex	SMA	8	−12	44	2.60	NA
Left precentral gyrus	M1a	−34	−20	52	2.51^#^	NA
Left SMA		−8	−14	48	2.49^#^	NA

# P < 0.05 (FWE-corrected) after small volume correction, NA: voxels not available within 5-mm sphere VOIs with sphere center at the coordinate in the iEMG corrected analysis.

**Figure 2:**
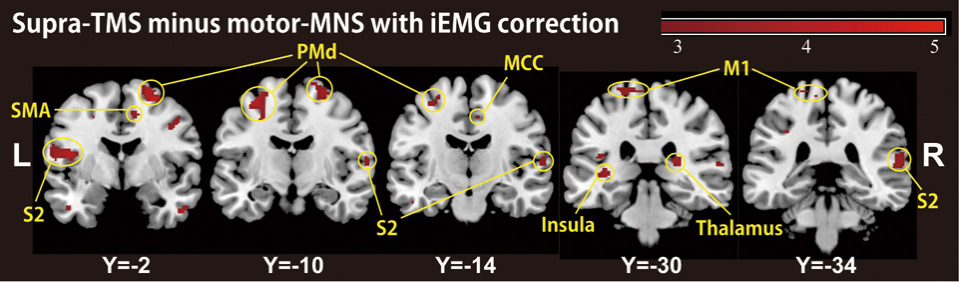
**A group-level statistical parametric map categorically comparing hemodynamic changes of the supra-TMS- and motor-MNS-induced activity after iEMG correction (*P* < 0.005 uncorrected for display purpose), corresponding to “pure” TMS-evoked activity**. This activity was observed in bilateral insula, bilateral secondary somatosensory area (S2), bilateral thalami, left primary motor cortex (M1), bilateral dorsal premotor cortex (PMd), supplementary motor cortex (SMA) and middle cingulate cortex (MCC).

### Localization of TMS-, muscle afferent- and superficial afferent-induced brain activity within M1 subdivisions

**Figure 3:**
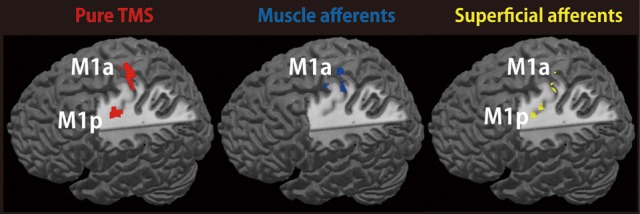
**Representations of TMS-, muscle afferent- and superficial afferent-induced brain activity within M1 subdivisions**. Muscle afferent-related brain activity was predominantly distributed in M1a while superficial afferent-related activity was seen in both M1a and M1p. Pure TMS-evoked activity was mainly distributed in M1a. *Red*, pure TMS-related brain activity [(supra-TMS minus implicit baseline) minus (motor-MNS minus sensory-MNS)]. *Dark blue*, muscle afferent-related brain activity (motor-MNS minus sensory-MNS). *Yellow*, superficial afferent-related brain activity (conjunction of motor-MNS and sensory-MNS). Activity was thresholded at *P* < 0.005 uncorrected for display purpose.

We assessed the spatial distribution of activated voxels representing “pure” TMS-, muscle afferent-, and superficial afferent-induced activity in the two subdivisions of M1, M1a and M1p (Figure 3). Visual inspection showed muscle afferent-related activity primarily in M1a and superficial afferent-related activity in M1p. To semi-quantify any biased distribution across the two MNS conditions, we calculated the M1-DI for the supra-threshold voxels representing muscle afferent- and superficial afferent-induced activity. The M1-DI for muscle afferent-induced activity (mean ± SEM, 0.30 ± 0.26) was significantly larger than that for superficial afferent-induced activity (0.01 ± 0.24) (*P* = 0.043 by Mann-Whitney *U* test). This indicates that muscle afferents were mainly represented in the M1a subdivision, while superficial afferents were distributed evenly across the two divisions. Similar DI analysis showed that “pure” TMS-evoked activity was predominantly distributed in M1a (0.32 ± 0.21).

### Amount of muscle afferent-induced activity included in supra-TMS-induced activity with and without correction for movement size

**Figure 4:**
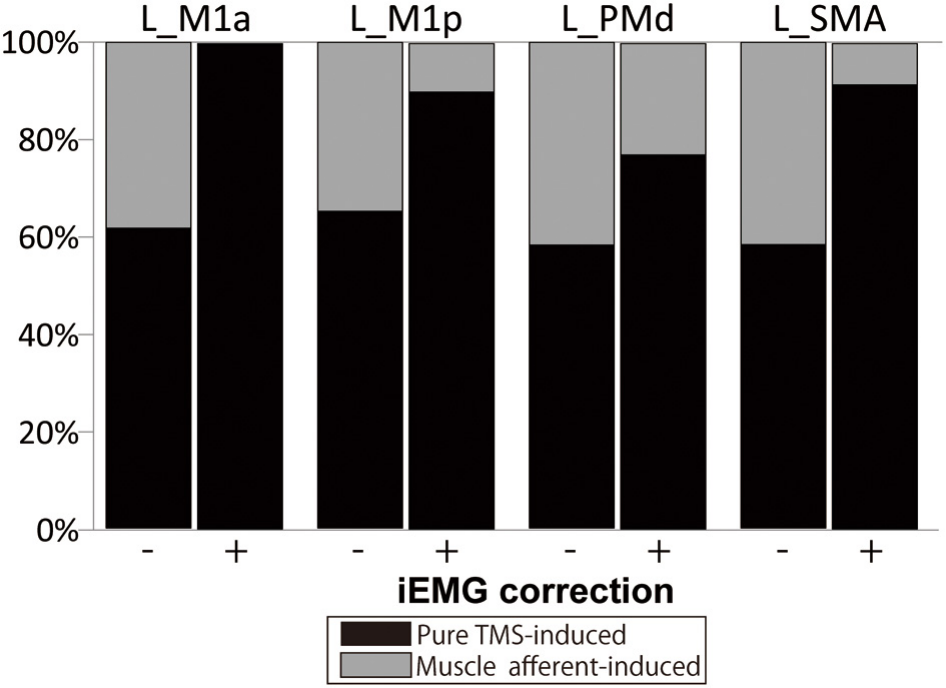
**Percentage of fMRI signals ascribed to the “pure” TMS- and muscle afferent-induced activity within supra-TMS-induced activity**. Data are shown for representative motor areas. *Black*, percentage of the contrast Supra-TMS minus Motor-MNS. *Gray*, percentage of the contrast Motor-MNS minus implicit baseline. (−), without iEMG correction. (+), with iEMG correction. *L*, left; *M1a* and *M1p*, anterior and posterior subdivisions of primary motor cortex; *PMd*, dorsal premotor cortex; *SMA*, supplementary motor area.

The muscle afferent-related responses included in the pure TMS-evoked responses without iEMG correction were around 40% in motor-related areas (Figure 4). When adjusted with the iEMG, motor-MNS-induced fMRI signals within supra-TMS-induced signals were 0, 10.0, 22.9, and 8.5% in left M1a, left M1p, left PMd, and left SMA, respectively. Thus, the present analysis indicates that “pure” supra-TMS components can explain approximately 80–90% of activity in motor areas during supra-TMS (120% RMT) delivered to M1.

## Discussion

The present study evaluated motor-area activity ascribed to deep and superficial sensory afferents in an effort to understand the properties of motor-area activity evoked by suprathreshold TMS delivered to M1. The present study used standard HRF to compare the size of TMS- and MNS-induced activities because previous evidence supported the use of standard HRF for detecting single-pulse TMS-induced and MNS-induced activities (Shitara et al., 2011). Distribution of cortico-cortico evoked activity in the motor network agreed with previous findings from simultaneous fMRI/PET studies (Bestmann et al., 2003; Fox et al., 2006; Hanakawa et al., 2009) and simultaneous EEG studies (Nikulin et al., 2003; Komssi et al., 2004; Fuggetta et al., 2005; Bonato et al., 2006) during TMS applied to M1. Additionally, the present MNS-induced brain activity was consistent with previous studies (Davis et al., 1995; Manganotti et al., 2009). Key novel findings here were: (1) muscle afferent-induced activity was mainly located in the rostral sector (M1a) of M1 also in humans and (2) “pure” TMS-induced activity in motor areas remained significant after removal of muscle afferent-induced activity and was located in both M1a and M1p.

### TMS intensity

In the present study, for participants who required stimulation above 100% of the machine output to reach the 120% RMT stimulation, the maximum machine output of the TMS stimulator was reset to 110% of the default machine output, within the safety guidelines of the manufacturer. No adverse effects were reported by the participants or observed by the investigators during or after the stimulation.

The mean RMT of 80.6% of machine output may seem high, considering TMS to M1 in standard, outside-MRI environments. There are a couple of reasons for this. First, we placed the TMS-coil orientation ~45° from the medial-lateral axis, but the handle of the TMS coil had to be placed toward the back because of the interference between the TMS coil and MRI head coil. This set-up induces currents in the posterior-to-anterior direction. With the bisphasic stimulation (Magstim Rapid), the RMT for the posterior-to-anterior current is higher than that for the anterior-to-posterior current (Kammer et al., 2001). Moreover, since we connected the MRI-compatible TMS coil to the TMS stimulator outside the radiofrequency shielded cabin via an 8-m cable, the TMS output intensities were supposed to be reduced by ~20% (Bestmann et al., 2003). We consider that these two factors primarily make RMT seemingly high in this simultaneous TMS-fMRI experiment. In fact, the mean RMT was 79.6 and 85.4% of the default machine output, respectively, in our previous TMS-fMRI studies (Hanakawa et al., 2009; Shitara et al., 2011). Hence, 80.6% of the machine output was not particularly high as for RMT in the simultaneous fMRI environment. In consistent, we observed MEP only in the right APB muscle although we recorded MEPs also from non-target muscles (right ADM and left hand). This finding indicated that TMS-evoked muscle activity did not spread to neighboring motor representations.

### TMS- or MNS-induced neural activities and fMRI signals

We are able to capture fMRI signal changes associated with short-lasting transient neural activity because fMRI measures slow haemodynamic changes (lasting several seconds after brief neuronal activations) as a surrogate marker of neural/synaptic activities (Friston et al., 1994). A proposed model to describe the temporal relationship between the neural/synaptic activities and vascular signals is called neurovascular coupling or HRFs. Because of the delays in hemodynamic responses, fMRI signal changes following neural/synaptic activities, which would last for only tens to hundreds of milliseconds after TMS- or MNS-stimulation, last over several seconds. To account for hemodynamic delay, convolution of modeled neural/synaptic activity changes with HRF is widely used to create regressors for fMRI data analyses. As we sampled such fMRI signals every 1 s, we were able to detect fMRI signal change correlated with neural/synaptic activity.

One may wonder if there are correlations in activity size between brief neuronal/synaptic activities and fMRI signals. Logothetis and colleagues have pioneered the simultaneous acquisition of electrophysiological and fMRI signal acquisition in primates. This work has shown that the fMRI signals are tightly coupled with electrophysiological activity, particularly local field potentials that represent synchronized synaptic inputs to a given neural population (Logothetis et al., 2001). This agrees with data showing a significant correlation between fMRI responses and evoked potentials in humans (Arthurs et al., 2000). Moreover, ample evidence from animal studies indicates correlations between short-lasting evoked field potentials and slow hemodynamic changes (Tsubokawa et al., 1980; Mathiesen et al., 1998; Brinker et al., 1999; Ngai et al., 1999; Nielsen et al., 2000; Ogawa et al., 2000). This justifies the “cognitive subtraction” method in which one may assume that motor-MNS condition would induce additional activity ascribed to muscle afferents on top of superficial sensory activity evoked by sensory-MNS.

### Effect of muscle afferents on supra-TMS-induced brain activity

Many simultaneous TMS-imaging studies have reported that stimulated M1 shows increased activity only with suprathreshold TMS (Bestmann et al., 2003; Fox et al., 2006; Hanakawa et al., 2009; Shitara et al., 2011). When different TMS intensities are applied, non-linear responses emerge in M1 around the motor threshold (Hanakawa et al., 2009), meaning that only supra-TMS accompanying MEPs induces activity in the region of M1 that is directly stimulated. One natural interpretation of these observations is that M1 activity during suprathreshold TMS may at least partially reflect proprioceptive afferents from twitched muscles, as discussed in previous reports (Bestmann et al., 2003; Komssi et al., 2004; Fox et al., 2006; Hanakawa et al., 2009). Previous evidence supports this “muscle afferent” hypothesis. Magnetoencephalography (MEG), intracranial electrical recording, and fMRI studies have consistently revealed M1 activity during MNS-induced muscle twitching (Spiegel et al., 1999; Huang et al., 2000; Balzamo et al., 2004). Furthermore, M1 plays essential roles in motor perception based on the processing of muscle-afferent information (Naito et al., 2002). Here, results partially support the idea that muscle afferents affect motor-area activity during supra-TMS applied to M1. We found widespread muscle afferent-induced activity in motor areas even though the experimental setup should have minimized their effects. In M1, moderate activity was observed during motor-MNS after removing the effects of sensory-MNS that induced only superficial sensory perception. Because motor-MNS induced activation in remote motor areas other than M1, muscle afferent-induced activity may even explain activity in the remote motor network during supra-M1 stimulation. Previous research has shown that MNS above the motor threshold can evoke long-latency cortical potentials, but not short-latency potentials, in motor-related areas such as the SMA (Allison et al., 1991). Although a wide representation of muscle-afferent information in motor-related areas is already recognized, previous neuroimaging studies have largely neglected the contribution of muscle afferents to motor-area activity during movement.

Here, we carefully examined the effect of muscle afferents on motor-area activity during supra-TMS. A simple comparison of activity between supra-TMS and motor-MNS revealed motor-area activity, strongly indicating that neither local nor remote activity induced by supra-TMS could be explained solely by muscle afferents. However, this finding was difficult to interpret, especially quantitatively, because of the significant differences in the amount of muscle activity (iEMG) across conditions. We therefore re-analyzed the data correcting for muscle afferent-induced activity using iEMG. The effects of muscle afferents on supra-TMS-induced activity can never be neglected. In M1, ~10% of activity during supra-TMS was ascribed to muscle afferents. Nevertheless, the analysis of supra-TMS-induced activity after removal of muscle afferent-related activity supports the claim that supra-TMS evoked neuronal and/or synaptic activity. Surplus activity during supra-TMS that is greater than the muscle-afferent component would include M1 neural activity involving both inter-neurons and pyramidal neurons to produce a significant number of descending volleys to the spinal motoneuron pools (Barker et al., 1985; Hallett, 2007). Although the small contribution of muscle afferent-induced activity to supra-TMS induced-activity is not surprising, previous TMS-fMRI studies only discussed this possibility. To our knowledge, the present study was the first to quantify contribution of muscle afferent-induced activity to supra-TMS induced activity. Since the muscle afferent contribution to supra-TMS-evoked motor network activity was relatively modest, we considered that it would be reasonable to use PET/fMRI with supra-TMS to M1 as a technique for evoked motor network imaging.

### Localization of the TMS-, muscle afferent- and superficial afferent-induced brain activity within M1 subdivisions

The findings here support the existence of two M1 subdivisions (M1a and M1p) in humans. Human thumb movements have been shown to be dually represented within these two subdivisions, each with a specific though not exclusive function (Geyer et al., 1996). M1a is considered to be “executive” and its activity directly results in actual movement. In contrast, M1p appears to be involved in a number of cognitive tasks and non-executive functions. M1p is activated by sensory inputs, modulated by attention (Binkofski et al., 2002; Johansen-Berg and Matthews, 2002), and affected by ageing (Ward and Frackowiak, 2003).

The rostral region of M1 (M1a) in squirrel monkeys primarily receives deep sensations from muscles and tendons, while the caudal region (M1p) receives superficial sensations from the skin (Strick and Preston, 1982). Consistently in other single-unit recording studies in non-human primates, the rostral M1 (M1a) predominantly receives non-cutaneous inputs while the cutaneous input is primarily confined to the caudal part of M1 (M1p) (Tanji and Wise, 1981; Picard and Smith, 1992). To our knowledge, no studies have shown detailed localization of TMS-, muscle afferent- and superficial afferent-induced brain activity within M1 subdivisions. The present results support the notion that in humans, muscle afferent-induced activity is localized primarily in M1a, although superficial afferent-induced activity is located in both M1a and M1p, despite being modest in intensity. Wide representations of superficial sensory inputs in humans could be related to dexterity of hand movements. Here, the supra-TMS condition did not include cognitive or attentional components and it should not evoke much activity in M1p. Another simple explanation for the M1a-predominant supra-TMS induced activity may be that M1a is closer to the scalp than M1p, and thus is more susceptible to TMS.

By capitalizing on superior temporal resolution, previous single-pulse TMS-EEG studies identified temporal evolution of TMS-evoked cortical activities. In addition to early activation of the stimulated and contralateral motor cortex (Komssi et al., 2002, 2004), several TMS-EEG studies have reported subsequent activation of frontal regions that likely coincide with ipsilateral supplementary/premotor areas (Paus et al., 2001; Komssi et al., 2004; Bonato et al., 2006; Litvak et al., 2007). It is not entirely clear if these late frontal activations reflect direct cortico-cortical evoked activity only or include effects of somatosensory afferents.

Suprathreshold TMS to the motor cortex results in muscle activations measured as MEP, accompanying muscle contraction and possibly joint movement. Such peripheral reactions should yield somatosensory afferents especially from muscles and joints and may generate somatosensory-evoked potentials detectable with EEG (Paus et al., 2001; Schürmann et al., 2001; Komssi et al., 2002; Nikulin et al., 2003). The early components of the TMS-evoked potential (<40 ms) are unlikely to be affected by somatosensory-evoked potentials, taking into account the conduction time from the cortex to the muscle and back again (Paus et al., 2001; Komssi et al., 2002). Evidence is available to argue for an idea that later components such as N45 and N100 may also be independent of sensory afferents as their peak amplitudes do not correlate with MEP size and they even occur at sub-threshold intensities (Paus et al., 2001; Nikulin et al., 2003; Komssi et al., 2004, 2007). Although these studies can obviously claim the presence of cortico-cortical potentials evoked by TMS, possible somatosensory components in the suprathreshold TMS-evoked EEG potentials have not been well specified.

Esser and colleagues reported that the early peaks of the global mean field power (<40 ms after suprathreshold TMS to M1) corresponded to activity in the ipsilateral M1-premotor cortex and that the later peaks (>40 ms after stimulation) corresponded to activity in the ipsilateral M1-S1 (Esser et al., 2006). They interpreted that later peaks included somatosensory components of MEP, and suggested the location of “pure” TMS-evoked activity was located more anteriorly than that of the sensory afferents induced activity. Ferreri and colleagues described that, in addition to N7 that likely reflected cortico-cortical TMS-evoked potentials in the premotor cortex, N44 might be related to somatosensory evoked potentials generated by TMS-induced muscles twitches (Ferreri et al., 2011). N44 showed a diffuse spatial distribution with antero-posterior amplitude gradient. This study indicated that “pure” TMS-evoked activity was distributed in M1 and premotor cortex and muscle/superficial afferent induced activities were diffusely located. These two TMS-EEG studies indicate that the comparison with somatosensory-evoked components can better characterize “pure” TMS evoked potentials. In reference to these TMS-EEG findings, we consider that the present study has advanced understanding of TMS-evoked motor network activity. By taking advantage of superior spatial localization, we showed precise localization of “pure” TMS-evoked activity in comparison with superficial/deep sensory mapping of motor areas. This information cannot be obtained with TMS-EEG only. We propose that TMS-EEG and TMS-fMRI are complementary methods, which together propel the understanding of motor network.

To interpret the relation between the superficial afferent and the muscle afferent, we referred to previous reports using cutaneous anaesthesia. Intriguingly, anesthesia of fingers induces short-term enlargement and spatial shifts of the cortical representation of the unanesthetized fingers (Rossini et al., 1994b). When the target muscle was totally “enveloped” within the anesthetized area but was still dispatching a normal proprioceptive feedback by temporal cutaneous block, the cortical representation of the target muscle was significantly reduced (Rossi et al., 1998). Moreover, variability in MEP and F-wave significantly decreased in the target muscle. Thus, those anesthesia studies have shown that the disruptions of superficial afferents induce modifications of the corticospinal pathways and somatotopical representations, suggesting complex interactions among superficial and deep sensory inputs, and motor representations. However, it should be noted that these complex processes most likely result from short-term neuroplasticity after anesthesia interventions. Although these plasticity issues are interesting, we should also possess basic knowledge of superficial and deep sensory representations in motor cortex without influence of plastic changes. For example, although we had previously measured fMRI activity induced by suprathreshold TMS (Hanakawa et al., 2009; Shitara et al., 2011), contribution of muscle twitch-induced sensory activity to the induced fMRI activity was unknown. Presence of plastic changes would make the interpretation of the findings difficult. We hence employed a simple approach by comparing fMRI activities across supra-TMS, motor-MNS and superficial-MNS conditions. This approach should provide a fundamental knowledge about sensory representations in the motor areas, without ongoing interactions between the motor commands and sensory afferents as seen in object manipulation or by short-term plasticity after anesthesia.

### Study limitations

In the present study, we contrasted the supra-TMS condition with the two MNS conditions, not with other TMS conditions. The effects of TMS to M1 could be non-specific such as eye blinking, tactile sensation on the scalp and loud auditory clicks (Jahanshahi and Rothwell, 2000; Anand and Hotson, 2002). Sounds induced by TMS may influence M1 since large sounds temporary suppress M1 excitability (Furubayashi et al., 2000). However, it is disputable if the TMS-induced click sounds significantly activates M1 during TMS-fMRI experiments. Hart et al. reported that activity in motor areas did not change in relation to sound levels (Hart et al., 2003). In contrast to the auditory cortex showing activity in proportion to sound levels, M1 activity changes abruptly at around the RMT (Hanakawa et al., 2009). Thus, it seems less likely that motor area activity in the present experiment was significantly affected by the levels of click sound produced by TMS. Similarly, we cannot completely exclude the possibility that tapping sensation of the scalp associated with TMS since it is exaggerated in the MRI environment even with careful fixation of the coil. The overlapping of the supra-TMS-induced activity with the MNS-induced activity strongly supports that the present supra-TMS-induced activity was localized to hand representation of the M1. However, the non-specific effects of TMS in the concurrent TMS-fMRI set-up need to be addressed formally in the future studies.

Finally, we applied peripheral nerve stimulation as control conditions for muscle afferents during supra-TMS stimulation to M1 and for superficial afferents. Since they are not natural somatosensory stimuli, it is uncertain if we can extend the present observations to the case of somatosensory stimuli associated with natural movements on one hand. On the other, we consider the MNS conditions have best served as control conditions to address somatosensory effects associated with supra-TMS as addressed here.

## Conclusion

Using simultaneous measurements from fMRI, TMS/peripheral nerve electrical stimulation, and EMG, our results provide the first evidence for detailed localization of TMS-, muscle afferent- and superficial afferent-induced brain activity within human M1 subdivisions. Muscle afferent-induced activity was mainly located in the rostral M1. Moreover, the present study demonstrated relatively limited effects of muscle afferents on supra-TMS-evoked local and remote activity in the motor network. Considering this, the present findings favor the interpretation that recruitment of neural populations contributes to motor-area activity during supra-TMS applied to M1. Based on these and previous findings (Hanakawa et al., 2009), we propose that the non-linear emergence of brain activity at the stimulated site might be a useful marker for threshold-level TMS.

## Funding

This research was partly supported by grants from PRESTO, KAKENHI (20033030 and 20019041) and the Takeda Science Foundation to T. Hanakawa and by grants from Grant-in-Aid for Young Scientists (B) (12813501) to H. Shitara.

### Conflict of interest statement

The authors declare that the research was conducted in the absence of any commercial or financial relationships that could be construed as a potential conflict of interest.
